# Classifications of triple-negative breast cancer: insights and current therapeutic approaches

**DOI:** 10.1186/s13578-025-01359-0

**Published:** 2025-02-01

**Authors:** Ziqi Chen, Yumeng Liu, Minchuan Lyu, Chi Ho Chan, Meiheng Sun, Xin Yang, Shuangying Qiao, Zheng Chen, Sifan Yu, Meishen Ren, Aiping Lu, Ge Zhang, Fangfei Li, Yuanyuan Yu

**Affiliations:** 1 Institute of Systems Medicine and Health Sciences, School of Chinese Medicine, Hong Kong Baptist University, Hong Kong, SAR China; 2 Guangdong-Hong Kong-Macao Greater Bay Area International Research Platform for Aptamer-Based Translational Medicine and Drug Discovery, Hong Kong, SAR China; 3 Institute of Integrated Bioinformedicine and Translational Science (IBTS), School of Chinese Medicine, Hong Kong Baptist University, Hong Kong, SAR China; 4 Law Sau Fai Institute for Advancing Translational Medicine in Bone & Joint Diseases, School of Chinese Medicine, Hong Kong Baptist University, Hong Kong, SAR China; 5 Key Laboratory of Animal Diseases and Human Health of Sichuan Province, College of Veterinary Medicine, Sichuan Agricultural University, Chengdu, People’s Republic of China

**Keywords:** Triple-negative breast cancer, Classification, Subtype, Tumor heterogeneity, Neoadjuvant therapy

## Abstract

Triple-negative breast cancer (TNBC) is an aggressive and challenging type of cancer, characterized by the absence of specific receptors targeted by current therapies, which limits effective targeted treatment options. TNBC has a high risk of recurrence and distant metastasis, resulting in lower survival rates. Additionally, TNBC exhibits significant heterogeneity at histopathological, proteomic, transcriptomic, and genomic levels, further complicating the development of effective treatments. While some TNBC subtypes may initially respond to chemotherapy, resistance frequently develops, increasing the risk of aggressive recurrence. Therefore, precisely classifying and characterizing the distinct features of TNBC subtypes is crucial for identifying the most suitable molecular-based therapies for individual patients. In this review, we provide a comprehensive overview of these subtypes, highlighting their unique profiles as defined by various classification systems. We also address the limitations of conventional therapeutic approaches and explore innovative biological strategies, all aimed at advancing the development of targeted and effective therapeutic strategies for TNBC.

## Background

Triple negative breast cancer (TNBC) accounts for approximately 10% to 20% of all breast cancer cases, with notable characteristics among women who are young (under 40 years old) [1], African-American, premenopausal, and from low socioeconomic backgrounds [2–4]. According to multivariable analysis, the incidence of TNBC in the USA was reported to be 13.7 cases per 100,000 women in 2020 [4]. In a cohort study conducted in Singapore, which included Chinese, Malaysian, and Indian populations, found a TNBC prevalence of approximately 13% [5]. TNBC is associated with a high likelihood of recurrence and distant metastasis, leading to lower survival rates. Nearly 40% of TNBC patients at stage I–III experience recurrence within the first 2 to 3 years after standard treatment [6]. The average overall 5-year relative survival rate of TNBC patients is 77%, which is 8% to 16% lower than that for hormone receptor-positive breast cancer [7]. However, it is well established that TNBC encompasses a variety of molecularly distinct tumors. Consequently, treatment plans are now customized not only based on the stage of the disease but also on the specific molecular subtype of the tumor [8].

Clinically, the major diagnostic methods for TNBC include mammography, ultrasound, magnetic resonance imaging (MRI), biopsy and histopathological analysis. Unfortunately, mammography screening is not very sensitive for detecting TNBC, with an accuracy of only 39.8% [9]. Ultrasound provides limited utility, primarily distinguishing between benign and solid lesions, but it falls short in identifying for malignant features [10]. While MRI is more sensitive, biochemical testing remains essential for cross-check, optimizing treatment [11] and predicting prognosis [12]. Among these methods, biochemical detection has the potential for early screening and precise treatment on a larger scale and at a lower cost.

The lack of a standardized subtyping method for TNBC complicates screening, detection and treatment strategies, while also limiting the applicability of existing clinical trial outcomes to a small subset of patient characteristics, thereby exacerbating the challenges of non-responding patients [13]. This situation adds to the inherent complexities of understanding TNBC. Furthermore, the absence of definitive biomarkers for elucidating mechanisms, precise subtyping, and addressing suboptimal responses to checkpoint inhibitors underscores the urgent need to deepen our understanding of TNBC. Gaining this insight is essential for advancing more personalized and effective therapeutic strategies [2, 5, 6].

The classification of TNBC is an ever-evolving field, as emerging insights from ongoing research are progressively integrated into clinical practice. Understanding the divergence between the comprehensive molecular classifications developed in research and the practical approaches adopted in clinical settings is crucial for improving treatment strategies for TNBC patients. In preclinical research, molecular subtyping [14], integrative approaches [15] and advanced techniques [16] are current methods to classify TNBC, while immunohistochemistry (IHC) remains the primary method applied in clinical practice [7, 9]. Molecular subtyping, using techniques such as RNA sequencing and gene expression microarray analysis, is widely employed to identify detailed molecular profiles [15, 17, 18]. Integrative analysis combines transcriptomics, proteomics, and epigenomics to identify consensus from heterogeneity [19, 20]. The latest techniques, including single-cell RNA sequencing and whole-genome sequencing (WGS), offer enhanced insights, although their clinical application is still in its early stage [11]. The development of TNBC classification methods highlights the importance of integrating research findings into clinical practice, which in turn facilitates a more precise categorization of TNBC subtypes and their therapeutic implications.

In this review, we aim to provide a comprehensive overview of these subtypes, highlighting their unique profiles as defined by various classification systems. We also examine the limitations of conventional therapeutic modalities and explore innovative biological approaches, all with the goal of advancing the development of targeted and effective therapeutic strategies for TNBC.

## Current clinical practice guidelines

**Fig. 1:**
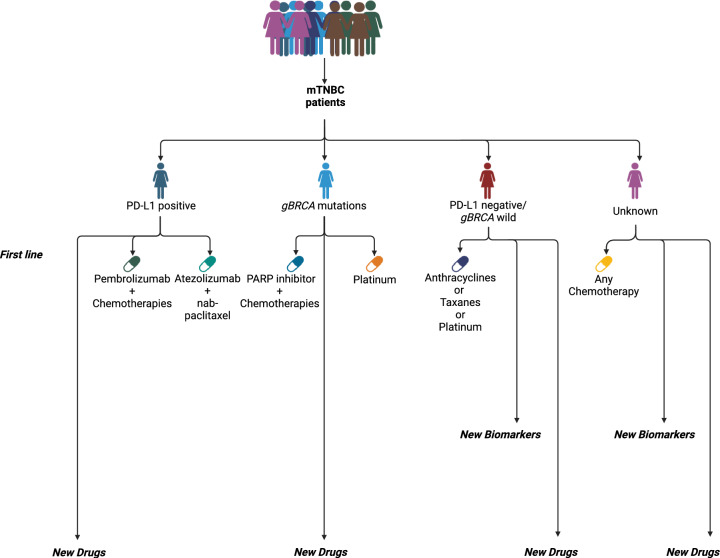
Consensus guidelines for TNBC treatment. This figure illustrates the biomarker-driven approaches for managing mTNBC as outlined in various clinical practice guidelines. It emphasizes the role of PD-L1 expression and *gBRCA* mutations as critical predictive biomarkers, reflecting a shared consensus among leading guidelines in optimizing treatment strategies for mTNBC

The National Comprehensive Cancer Network (NCCN) guidelines recommend assessing PD-L1 expression and *BRCA* mutations in TNBC patients to guide the initial therapeutic decisions (Fig. 1). Treatments are subsequently adjusted based on disease progression, which involves monitoring tumor dynamics and metastatic status, yet further TNBC subclassification is not pursued [21]. Similarly, the European Society for Medical Oncology (ESMO) guidelines provide an evidence-based framework for TNBC management, also prioritizing PD-L1 and germline *BRC*A (*gBRCA*) biomarkers. However, for patients who test negative for both PD-L1 and *gBRCA*, the Magnitude of Clinical Benefit Scale does not recommend any first-line targeted therapies [22]. This broad-spectrum approach may pose the risk of inappropriate or unnecessary treatments for patients who do not respond to initial therapy due to its limited precision. Furthermore, it may lead to the oversight of nuanced clinical presentations, potentially leading to delayed or overlooked diagnoses. Beyond PD-L1, the Chinese Society of Clinical Oncology (CSCO) guidelines and the World Health Organization (WHO) classification also recommend reporting on *Ki-67* and tumor-infiltrating lymphocytes (TILs) as additional markers to better determine the molecular characteristics of the tumor.

## Systematic classifications and therapeutic approaches

To navigate the complexities of heterogeneity in TNBC and tailor optimal treatments for patients, several systematic classification strategies have been established [17, 23–30] (Table 1). These approaches involve a comprehensive analysis of genetic variations, including both inherited factors and spontaneous mutations, as well as the examination of transcriptomic differences. Additionally, they focus on identification of biomarkers that are essential for modulating key cellular processes, such as kinase activity, cell cycle regulation, apoptosis, and DNA repair mechanisms.

**Table 1:** Categories of TNBCs based on data sources and subtype characteristics

Category	Data sources	Subtypes
Cellular biological features		
Metastatic ability		Metastatic TNBC (mTNBC); Solidary (in situ) TNBC
Invasive ability		Preinvasive TNBC; Invasive TNBC
Molecular features		
Origin of mutation		Somatic TNBC; Germline TNBC
PAM50 [23]	IHC, Transcriptomic analysis	Luminal A, Luminal B, Normal-like, HER2-enriched and Basal-like
CNA characteristic [24]	Gene Expression	Amplification (MYC, PIK3CA, CDK6, MDM2); Deletion (MAP2K4, TP53, NCOR1, BRCA, PTEN, INPP4B)
Metabolic pathway [25]	Mass Spectrometry on cell lines	MPS1 TNBC (lipogenic subtype with upregulated lipid metabolism) MPS2 TNBC (Glycolytic subtype with upregulated carbohydrate and nucleotide metabolism) MPS3 TNBC (Mixed subtype with partial pathway dysregulation)
Burstein classification[26]	Genomic and Transcriptomic analysis from patient tumors	Luminal Androgen Receptor (LAR), Mesenchymal (M), Basal-like Immunosuppressed (BLIS), and Basal-like Immune Activated (BLIA)
Polar metabolite and lipid profiling [27]	Genomic, transcriptomic from whole exome sequencing	Metabolomic C1 tumor: Sphingolipids and fatty acid enrichments Metabolomic C2 tumor: Upregulated carbohydrate metabolism and oxidation reaction Metabolomic C3 tumor: Mild metabolic differences
FUSCC classification [28]	IHC; Genomic and Transcriptomic from patient whole exome sequencing	Immunomodulatory (IM); Luminal Androgen Receptor (LAR); Mesenchymal subtype (MES); Basal-Like immunosuppressed subtype (BLIS);
Immune effectors (CK5 antibody) [29]	IHC; Genomic and Transcriptomic from patient DNA chip	C1: luminal subtypes and Luminal Androgen receptor (LAR) C2: Almost Basal-Like cluster C3: Basal-Like subtypes (BLS), 26% of claudin-low subtypes
Lehmann classification [17]	Histopathological, genomic, transcriptomic	Basal-like 1/2 (BL1/2); Immunomodulatory (IM); Mesenchymal (M); Mesenchymal Stem-Like (MSL); Luminal Androgen Receptor (LAR); Unstable (UNS) Mesenchymal (M), Immunomodulatory (IM), Luminal Androgen Receptor (LAR), BL including BL1 and BL2
Resistant to immune checkpoint inhibitors [30]	FACS and analysis on murine mammary tumor model	Neutrophil-enriched (NES); Macrophage-enriched subtype (MES)

### PAM50 classification

The PAM50 assay, which assesses a group of 50 transcriptomic factors, provides an intrinsic subtyping system that, while not limited to TNBC, commonly categorizes TNBC cases as basal-like (BL) subtypes due to their gene expression profiles [31, 32]. Through PAM50 analysis, the risk of distant metastasis in breast cancer, measured by a recurrence score ranging from 0 to 100, can be determined by analyzing the distribution of molecular subtypes and proliferation indices, which are then integrated with statistical weights. Traditionally, PAM50 classifies breast cancer into Luminal A, Luminal B, Normal-like, HER2-enriched and BL subtypes (the latter being commonly associated with TNBC), by comparing the expression of a set of 10 representative genes out of the 50 genes for each subtype [32]. However, discrepancies have been observed when PAM50 classifications based on single-gene expression scores for ESR1, PGR, and ERBB2 are compared to their corresponding IHC markers, resulting in misalignments that do not accurately reflect prognostic outcomes [23, 33]. In the GEICAM/CIBOMA trial, adjuvant therapy with capecitabine were recommended for PAM50 non-basal patients over PAM50-basal with early-stage TNBC (hazard ratio 0.19 verse 0.90) [34].

### Lehmann classification

In 2011, Lehmann et al. classified TNBC into six distinct subtypes primarily based on gene expression profiles, with validation through IHC across various datasets [18]. This initial classification, known as TNBCtype, was developed to evaluate the effectiveness of subtypes in relation to standard treatments by attributing similarities among them [17, 18]. The six subtypes include immunoregulation (IM), mesenchymal stem-like (MSL), mesenchymal (M), basal-like 1 (BL1) and basal-like 2 (BL2) and luminal androgen receptor (LAR). Within this framework, Lehmann proposed that pharmacologically targeting the predicted “drivers” in each signaling pathways could lead to distinct therapeutic selections. Furthermore, the exploration of the IM and MSL subtypes has provided substantial insights into the characteristics of M subtype. For instance, the cellular morphology of IM and MSL subtypes predominantly exhibits a spindle-like shape in two-dimensional cultures, along with lower proliferation rates. The MSL subtype is associated with epithelial-mesenchymal transition (EMT) through pathways such as TGFβ, ECM-receptor interaction, ALK, and Wnt/β-catenin. The IM subtype is distinguished not only by its unique immune features but also by the presence of interferon regulatory factors and tumor necrosis factor (TNF) [17, 18], which are fundamental in explaining the heterogeneity observed within the M subtype [17, 18]. In terms of drug response, the BL1 and BL2 subtypes preferentially respond to cisplatin due to higher expression levels of cell cycle and DNA damage response genes, while the M and MSL subtypes respond to phosphoinositide 3-kinases (PI3K)/mammalian target of rapamycin (mTOR) inhibitor. The LAR subtype, on the other hand, shows decreased relapse-free survival (RFS) when treated with androgen receptor (AR) inhibitors [18]. Overall, the TNBCtype classification represents the first systematic classification approach to categorizing TNBC and has significantly advanced our understanding of TNBC heterogeneity and its therapy implications.

Later in 2016, Lehmann’s team improved their classification system. They identified a significant association between transcriptomic profiles and clinical features, which has important implications for guiding the selection of targeted treatments [17]. Therefore, they categorized TNBC patients into four subtypes, including M, LAR, BL1 and BL2, collectively referred to as TNBCtype-4. The M subgroup was expanded to include MSL and IM, characterized by the overactivation of similar traits within TNBCtype. This expansion added clinical predictions, such as a higher propensity for aggressive lung metastasis, which is associated with the lowest overall survival (OS) and RFS rates. These poor outcomes are partly attributable to a desert-like immune phenotype [17]. The M subtype is characterized by sarcoma-like or squamous epithelial cell-like features, also known as metaplastic breast cancer [33]. Lehmann highlighted correlations among various indicators, noting that pathways such as Wnt, ALK, Rho are predominantly involved in maintaining a dedifferentiated state through EMT. EMT further promotes the proliferation of cancer stem cells (CSCs), which play a critical oncogenic role, especially in the MSL subtype [33]. CSCs represent a distinct subset of cancer cells with unique properties, and targeting receptors and/or pathways that are dysregulated in CSCs is a promising strategy for developing targeted therapies [35]. For patients with M subtype TNBC, clinical recommendations include considering inhibitors of the mTOR as part of neoadjuvant treatment [33, 36]. PI3K pathway, an upstream regulator of mTOR, has inhibitors that demonstrated efficacy in targeting metaplastic subtypes. Additionally, sonidegib, which targets the hedgehog pathway, has been shown to decrease fibrillar collagen, thereby enhancing the efficacy of docetaxel and potentially improving the clinical benefit rate (CBR) for patient to 25% [37].

LAR subtype is characterized by the presence of ARs, and hormone-regulated pathways, exhibiting features of apocrine differentiation, such as high or positive expression of luminal cytokeratins, frequent genetic mutations in *PIK3CA* or *AKT*, and low or absent basal cytokeratins and Ki-67 expression. Notably, a higher prevalence of the LAR subtype has been observed among Asian women [33]. Clinically, LAR cases are predominantly lobular carcinomas and are more likely to metastasize to lymph node and bones [17, 38]. The LAR subtype is more sensitive to anti-AR therapies and traditional anti-estrogen therapies, demonstrating lower rates of pathological complete response (pCR) but improved OS, particularly in AR-positive patients following neoadjuvant chemotherapy [39]. In the context of targeted therapies, AR-positive patients within the LAR subtype benefit from a broader range of treatment options and superior outcomes compared to AR-negative patients. For example, bicalutamide, an AR antagonist, has shown increased specificity for LAR cell lines [40]. Enobosarm combined with pembrolizumab has exhibited modest efficacy in AR-positive metastasis TNBC (mTNBC), with a notable CBR compared to pembrolizumab monotherapy (25% vs 12%) [41]. The combination of enzalutamide and taselisib has achieved a CBR of 35.7% in evaluable patients [42]. Moreover, the efficacy of PI3K/AKT inhibitors in LAR is also notable, with a CBR of 75%, starkly contrasting with the 12.5% observed in the non-LAR groups [43]. However, in AR-negative patients, these therapeutics exhibit restricted efficacy. Ongoing research has identified ACSL4, SKP2, EGFR, and CD151 as potential therapeutic targets, with ACSL4 and SKP2 also showing potential as biomarkers for this subtype [44].

Nearly 75% of TNBC is classified as BL. This subtype is further divided into two distinct groups: BL1 and BL2. The BL1 subtype demonstrates increased activity in growth factor signaling, cell cycle progression, and DNA damage regulation pathways, with genetic alterations that include amplifications in *MYC*, *PIK3CA* and *CDK6,* as well as deletions in *BRCA2, PTEN*, *MDM2*, *RB1*, and *TP53* [45]. In contrast, BL2 is characterized by abundant growth factor signaling and the presence of myoepithelial markers, including EGFR, MET, TGFβ, Wnt/β-catenin, and IGF-1R [17]. Both BL1 and BL2 subtypes show increased sensitivity to cisplatin compared to other subtypes [45]. Specifically, the BL1 subtype responds favorably to treatments with PARP inhibitors and genotoxic agents that target DNA repair mechanisms. For the BL2 subtype, clinical recommendations include the use of mTOR inhibitors and growth factor inhibitors [33]. Although BL2 typically presents a lower pCR rate, it is associated with a decreased rate of cancer recurrence following neoadjuvant chemotherapy [40].

The Lehmann classification includes the most subgroups, indicating initial treatment tendencies and predicting medication responses. This classification emphasizes the hormonally regulated features with a preference for bone metastatic in the LAR subtype and low lymph nodes involvement with lung metastasis in the M subtype helping physicians understand the heterogeneity of TNBC and identify differences in prognosis across subtypes. Additionally, a distinctive feature of the Lehmann classification is that it generated each subtype based on patients who receive neoadjuvant chemotherapy [18]. The analysis also validated that the degree of TILs was positively correlated with the efficacy of neoadjuvant chemotherapy. BL subcategories and characterizations were the most prevalent in this classification. However, the Lehmann classification lacks insufficient immune subclassification and presents a mismatch when simplifying from six to four subtypes. Limited external validation has made horizontal comparisons challenging. Furthermore, this classification is primarily based on data from the TCGA and METABRIC databases, which may lead to issues such as sample selection bias and insufficient sample sizes for specific cancer types when applied in clinical settings.

### Burstein classification

In 2015, Burstein et al*.* reclassified a cohort of 198 TNBC patients into four distinct subgroups, including LAR, mesenchymal (MES), basal-like immunosuppressed (BLIS), and basal-like immune-activated (BLIA), utilizing PAM50 gene expression profiling and claudin-expression patterns [26]. The Burstein LAR subtype shows similarities to the Lehmann LAR classification but includes with a small number of BL subtype cases. LAR is distinguished not only by androgen-related genes, but also by frequent amplification of genes such as *CCND1*, *FGF* family, *MDGA2,* and deletions in *RAD17*, the *ERBB* family and *CCNT1*. Despite aberrant expression of estrogen-regulated genes (*PGR*, *FOXA*, *XBP1*, *GATA3*) and the estrogen receptor (ER) alpha-coding gene *ESR1*, LAR often scores as ER-negative in IHC due to unconventional expression patterns [26]. IHC features of LAR include the lower levels of stromal TIL and a lower Ki-67 labeling index compared to other subtypes. Clinically, LAR presents a prognosis comparable to BLIA subtype in terms of RFS. The occurrence of the LAR subtype is less likely in patients under the age of 50 [26, 46, 47]. Therapeutical strategies for the Burstein LAR subtype align with Lehmann LAR recommendations. For patients who are AR-negative and exhibit MUC1 overexpression, a MUC1 vaccine is advised in addition to AR antagonists [26].

The MES subtype is characterized by a distinctive overexpression of genes typically associated with osteocytes and adipocytes, along with the essential insulin-like growth factor 1 (IGF-1). This subtype is marked by significant cellular signaling related to cell cycle regulation, mismatch repair, and DNA damage response mechanisms. Pathologically, the MES subtype lacks luminal differentiation markers and is linked to a worse prognosis compared to other subtypes, indicative of a more stem-like phenotype. MES comprises MSL cells and a subset of claudin-low mesenchymal tumors. IHC analysis has identified reduced cell-to-cell adhesion and features of metaplastic and invasive lobular carcinoma, which are hallmarks of this subtype [26, 46, 48]. For the MES subtype, the potential therapeutic value of pathway-specific inhibitors, such as those targeting β-catenin, IGF, and PDGFR, have been highlighted, suggesting promise in treating this aggressive form of breast cancer [26, 46, 48].

The BLIS subtype is characterized by its gene expression profile, which mirrors that of BL cells, including the upregulation of various SRY-box (SOX) transcription factors [26]. Pathological analyses have revealed that the BLIS subtype exhibits a suppression of immune regulatory pathways involving B cells, T cells, and natural killer (NK) cells, as well as cytokine signaling pathways. This immunological deficit correlates with an increased tumor size, typically ranging between pT2 and pT3, and is linked to the poorest prognosis in terms of RFS among all subtypes [46]. Given the immunosuppressive nature of the BLIS subtype, therapeutic strategies focusing on modulating the immune response, such as the administration of PD-1 and VTCN1 antibodies, are recommended for clinical evaluation. These immunotherapies potentially counteract the intrinsic immune resistance of the BLIS subtype, offering a targeted approach to improve patient outcomes [46].

The BLIA subtype also shares BL cell signatures, but diverges significantly by upregulating immune regulatory pathways, including a distinctive activation of the STAT signaling pathway [26]. Tumors classified as BLIA are generally smaller in size (pT1) and exhibit a higher density of TILs, indicating immune engagement. This immune activation is associated with a more favorable prognosis for BLIA, which demonstrates the lowest rates of recurrence among BL subtypes. Given the active immune landscape of BLIA tumors, therapeutic interventions such as CTLA4 inhibitors, aimed at amplifying the anti-tumor immune response, and STAT pathway inhibitors tailored to the activated signaling in these tumors, show considerable promise. Additionally, therapies that modulate the immune environment, including those that involving cytokines or cytokine receptor antibodies, could also prove advantageous [26, 46].

The Burstein classification is more concise, with subtype names reflect their characteristics and treatment options, making clinical application simpler and easier to implement in practice. Since this classification predominantly derived its samples from clinical patients, it emphasizes clinical relevance. A major contribution of Burstein’s work is the division of immunophenotyping into immuno-active and immune-suppressive categories. Furthermore, the extensive external validation of Burstein’s subtyping facilitated easy comparison of similarities and differences among datasets. However, the relatively small sample size may affect the generalizability of the results. Additionally, the lack of detailed analysis on the relationship between elevated and reduced genes/proteins in each subtype and predictive analyses may limit its comprehensiveness. The classification also did not include information on whether patients had a history of medication or specific treatment modalities.

### Jézéquel classification

The Jézéquel group has incorporated a multifaceted analysis that includes the claudin-low type, immunological profiles, cellular functional tests, neurogenesis-related factors, and clinicopathological outcomes to classify TNBC into three clusters: cluster 1, 2 and 3 [29]. This comprehensive approach offers a comparative perspective against existing classification frameworks.

In Cluster 1 (C1), there is a strong correction in gene expression with the molecular apocrine subtype, particularly concerning the PI3K pathway. This feature aligns with the LAR subtype in Lehmann’s system [18, 43]. C1 is characterized by an enrichment of luminal signaling and a deficiency in BL signaling. It exhibits few significant bio-functional traits and the lowest metabolic activity, suggesting that it may represent a less aggressive form of the disease. However, this does not necessarily lead to improved event-free survival (EFS) or RFS outcomes [49], which could be attributed to the absence of immune cell involvement, as TILs are generally associated with a more favorable prognosis [50]. Additionally, C1 encompasses A and B subtypes (as per PAM50) that carry a more pessimistic prognosis. When compared to other classifications, C1 includes the LAR, M and BLI subtype from the Lehmann classification. It is important to note that the inclusion of data from HER2-positive breast cancer cases within the study may dilute the specificity of TNBC subtyping. Therapeutic strategies for C1 may include the use of antiandrogens and agents targeting the PI3K or ERBB2 pathways [17, 40, 41, 43].

Cluster 2 (C2) is characterized by low immune response and a high presence of M2-like macrophages, aligning with the BLIS subtype of Burstein classification [26]. Given that the BL cellular composition of C2, a more precise genetic discrimination based on gene ontology (GO) annotations for biological functions is advisable. C2 is distinguished by an elevated expression of genes associated with cell proliferation, including *MKI67*, *UBE2C*, and *RACGAP1*, along with reduced expression of genes linked to EMT, such as *CDH2*, *TGFB1*, as well as immune response genes including *CD4*, *CD79A*, *IL6*. Consequently, functional pathways involving E2F3, PNI, TGFβ, VEGF, and the YAP1-WWTR1 complex are notably active in C2. Immune cell profiling within this cluster revealed a predominance of cells that facilitate tumor growth rather than combat it. Presenting high immune suppressive, high neurogenesis (nerve infiltration), and high biological aggressiveness, C2 is associated with the poorest prognosis among the three clusters, offering limited therapeutic avenues. VTCN1 stands out as a potential immune target with the capability to directly suppressing T-cell-mediated immune responses. Furthermore, Jézéquel has proposed investigating anti-neurogenic therapies as a novel approach to curtail cancer progression in C2.

Different from C2, Cluster 3 (C3) comprises of BL cells and demonstrates superior immune attributes, as evidenced by various indicators, including gene expression profiles, signaling pathways, pathologic assessments, and a positive response to immunotherapy. C3 is particularly notable for its high reactivity to B cells and presents a state of rest in cytotoxic T cells, major histocompatibility complex classes I and II (MHC-1 and MHC-2), along with an overexpression of lymphangiogenic chemokines. A significant finding within C3 is the identification of at least 34 immune checkpoints, including CD274 (PD-L1), PDCD1 (PD-1), and the well-recognized CTLA-4, offering promising targets for therapy. Patients in C3 often experience the most favorable outcomes in terms of OS and EFS due to their enhanced anti-tumorigenic capabilities.

The Jézéquel classification subdivided TNBC into three types, placing special emphasis on immune-relative factors. The hallmark of this classification is the grouping of patients based on macrophage responses in the tumor microenvironment, which facilitates the evaluation of patient survival and treatment response. With data source came from TNBC patients who had not received treatment, this classification may represent a more accurate etiological analysis of the genome and transcriptome. Moreover, it briefly mentions the metabolism and potential side effects of each subtype. Jézéquel was the first to systematically suggest that targeting neurogenesis may be beneficial for the immune-suppressive subtype of TNBC. Comprehensive external validation data also support meaning comparisons. However, the Jézéquel subtype exhibits inconsistencies in cytomorphology and classification. For instance, the overall analysis lacks clear delineation of mesenchymal-like cells, leading to confusion when summarizing the characteristics of these cells. Additionally, C2 and C3 show high similarities in their immunological macroscopic classification sub-profiles, highlighting another instance of unclear distinction. Other limitations include a small sample size of 107 cases and the inability to provide suboptimal treatment options for patients already on medication who are not responding, as well as those with existing drug resistance.

### FUSCC classification

The Fudan University Shanghai Cancer Center (FUSCC) classification for TNBC is a recognized system tailored specifically for Chinese patients. It integrates transcriptome profiles of both mRNA and lncRNA to accurately characterize TNBC into IM, LAR, MES and BLIS subtypes [14, 28, 51]. The utility of this classification has been validated through radio-genomic analysis [52].

Aligned with the Lehmann classification, the IM subtype is delineated by an amplification in cytokine signaling, immune cell signaling and TILs. This subtype shows a marked activation of pathways associated with the adaptive immune system, including those related to interferon-gamma. Furthermore, both clinical and omics data have evidenced the upregulation of the immunosuppressive enzyme IDO1 within IM subtype. Despite the presence of this immunosuppressive marker, the use of immune checkpoint inhibitors targeting PD-1, PD-L1, CTLA-4, and IDO1 is recommended for the IM subtype, as these treatments have been linked to promising therapeutic outcomes [28].

The LAR subtype is highly enriched in hormonally regulated pathways, with significantly elevated androgen and estrogen metabolism, steroid hormone biosynthesis, porphyrin and chlorophyll metabolism, and PPAR signaling pathways. Compared to TCGA data, the FUSCC subtyping reveals a higher prevalence of the LAR subtype in Chinese patients (23% versus 9%). Upon examining the etiology of the condition, a small subset of LAR cases exhibited chromosomal instability, with approximately 1 in 33 cases linked to mutations that cause homologous recombination deficiency (HRD). Genetic alterations, particularly in *PIK3CA*, *PTEN*, and *PIK3R1*, show a significant correlation with the LAR subtype. Additionally, mutations in *HRAS* and *ERBB2* although less common, are observed in 2% of cases. A noteworthy frequency of deletions was observed in *CDKN2A* (65%), and the authors also confirmed a reduced expression of *CDKN2A* and *E2F3* transcripts, implicating their significance in cell cycle regulation. Beyond AR-targeted therapies, checkpoint inhibitors such as CDK4/6 inhibitors and other cell cycle inhibitors may offer promising therapeutic alternatives for the LAR subtype [51, 53].

In theory, patients with the BLIS subtype could potentially benefit significantly from established chemotherapy, such as doxorubicin and cyclophosphamide (AC), or docetaxel in combination with AC, as well as platinum-based compounds like carboplatin and PARP inhibitors. The potential for this benefit is partly due to the fact that 65% of mutations in the HRD cancers are present in the BLIS subtype [51]. On the other hand, patients who exhibit resistance to treatments aimed at HRD-associated cancers often have a poor prognosis [19, 51, 54]. Genomic profiling of these individuals tends to reveal a propensity for whole-genome doubling, a factor that complicates treatment, especially since targeted therapy options for this particular group within the BLIS population are currently insufficient.

Aligned with the MES subtype in Burstein classification and the M subtype in Lehmann classification, the FUSCC MES subtype encompasses a broad spectrum of genomic alteration and exhibits an intermediate genomic profile. Upon analyzing the gene mapping, the MES subtype displays feature reminiscent of CSCs. Critical pathways, particularly JAK/STAT3, have been the focus of extensive investigation. Analysis has revealed that key participants of the JAK/STAT3 pathway, including agonists such as JAK1 and IL6, as well as the pathways marker pSTAT3, are overexpressed in the MES subtype [14]. Therefore, STAT3 inhibitors are recommended for this subtype [14, 55].

Later, in 2022, the Fudan group further integrated metabolic profiling, including analyses of polar metabolites and lipids, into the classification system to subgroup TNBC into distinct metabolic subtypes. By combining previously established transcriptomic/genomic data with polar metabolome and lipidome profiles, they classified the same cohort of patients into three distinct metabolomic subgroups. Type C1 is distinguished by an abundance of sphingolipid and fatty acid metabolism, whereas Type C2 is associated with elevated carbohydrate metabolism and oxidative activities. Type C3 is identified by more nuanced metabolic variations [27]. Analysis of ceramide metabolism revealed that the C1 subtype overlaps with the LAR subtype and is characterized by abundant sphingolipids, suggesting sphingosine-1-phosphate (S1P) as a potential therapeutic target for LAR subtype. The C2 and C3 subtypes include the BLIS subtype, and their pathological mutations lead to overexpression of *N*-acetyl-aspartyl-glutamate (NAAG), indicating that targeting NAAG could be a feasible treatment strategy for both subtypes [27, 30].

The FUSCC classification differs from the models of Lehmann and Burstein by integrating comprehensive genomics with extensive clinical validation. It considers both clinicopathological and molecular tumor characteristics, enhancing its practical application in the clinic. For instance, although the BLIS subtype accounts for 65% of TNBC due to HDR deficiency, HDR scores should also be considered in treatment, indicating limited benefits from DNA repair-targeting therapies. Additionally, this subtyping helps predict the most suitable drug for patients, guiding clinical treatment protocols. However, like other classification systems, FUSCC has limitations, including potential selection bias from its focus on a Chinese population and reliance on transcriptome and signal transduction abnormalities, which may not fully capture the tumor’s biological features. Additionally, significant gaps remain between the metabolome classification [27] and the popular quadruple typing system [28]. This disparity limits the generalizability of metabolic drugs, such as glucocorticoid receptor drugs, regarding their efficacy and prognostic value.

### Correlations among four classification systems

**Fig. 2:**
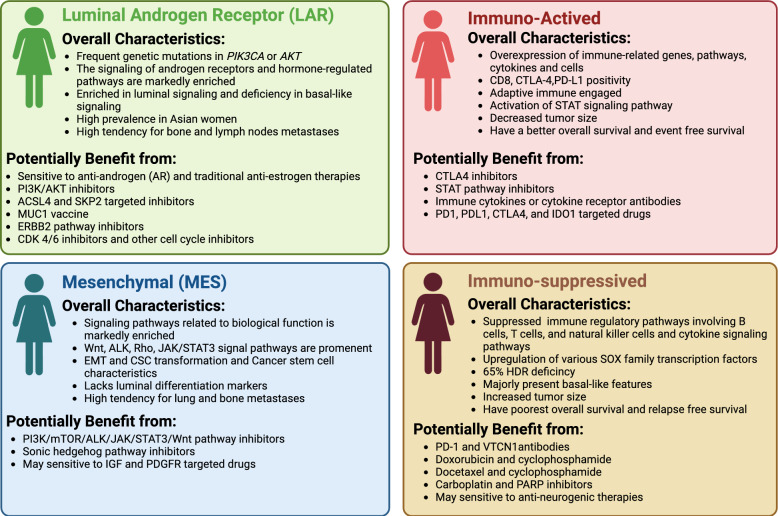
Characteristics of TNBC subtypes and associated treatment options. This figure summarizes four distinct characteristics of TNBC subtypes, along with their corresponding treatment options

We have summarized and compared the characteristics of four classification systems (Fig. 2 and Table 2).

**Table 2:** Summary of the four TNBC classification systems

Sub-items	Lehmann	Burstein	Jézéquel	FUSCC
Data source	TCGA	TCGA+ patients	TCGA+ patients	TCGA+ patients
Initial classification criteria	Transcriptional groups profiling	RNA and DNA genomic profiling	Therapeutic outcomes	Multiple-omic groups
Intentional Therapy	NACT	No	No	No
Subtype-relate therapy	Yes	Yes	Yes	Yes
External validation	Yes	Yes	Yes	Yes
Gene mutation	Yes	Yes	Yes	Yes
Pathways mutation	Yes	Yes	Yes	Yes
Metastasis preference	Yes	No	No	Yes
Metabolic characteristics	No	No	Yes	Yes
Histological differences	Yes	No	No	No
IHC validation	Predicted	Yes	Yes	Yes
Local clinical data	No	Yes	Yes	Yes
Adverse effects	Not given	Not given	Yes	Yes
Relative cell lines	Yes	No	No	Yes
Characteristics	More transcriptional; cellular details	More epigenetic analysis	More immune modulation details More external validation details	Asian patients Multi-omics data More mesenchymal-like details

**Fig. 3:**
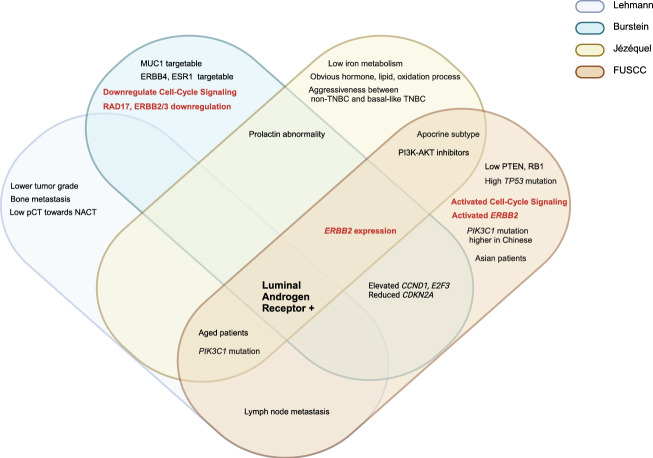
Consistency and differences in LAR characteristics. Venn diagram illustrates the consistency and differences among four TNBC classification systems regarding LAR subtype characteristics. Controversial points for the typologies are highlighted in bold brown

**Fig. 4:**
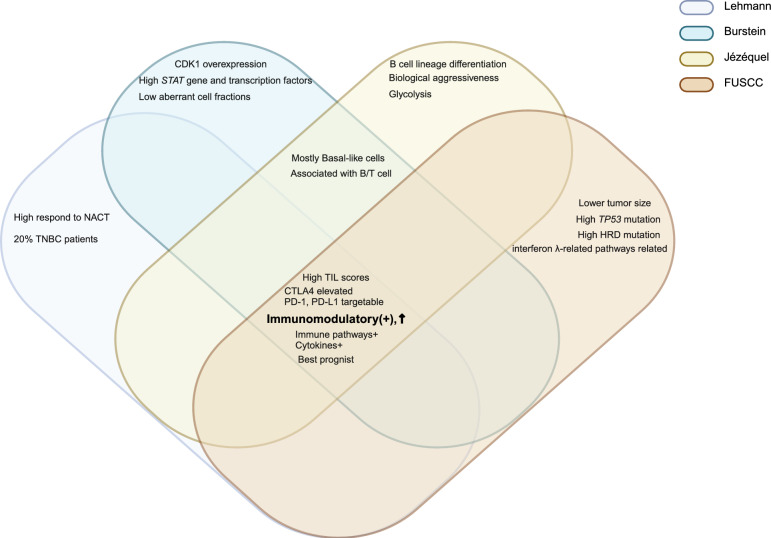
Consistency and differences in immunomodulatory characteristics. Venn diagram illustrates the consistency and differences of four TNBC classification systems regarding immunomodulatory active characteristics

**Fig. 5:**
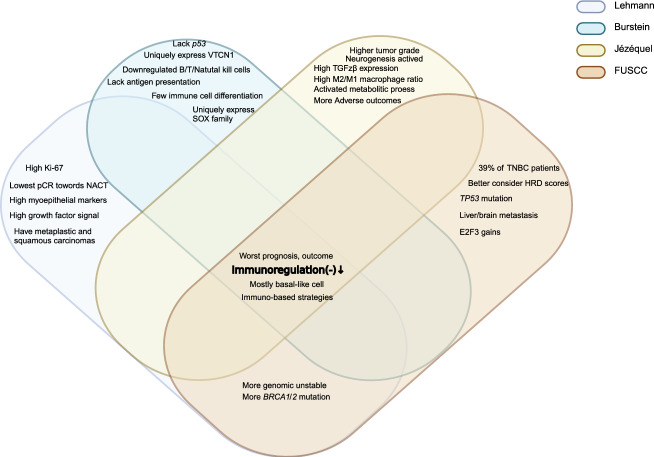
Consistency and differences in immunomodulatory characteristics. Venn diagram illustrates the consistency and differences of four TNBC classification systems regarding immunomodulatory suppressive characteristics

**Fig. 6:**
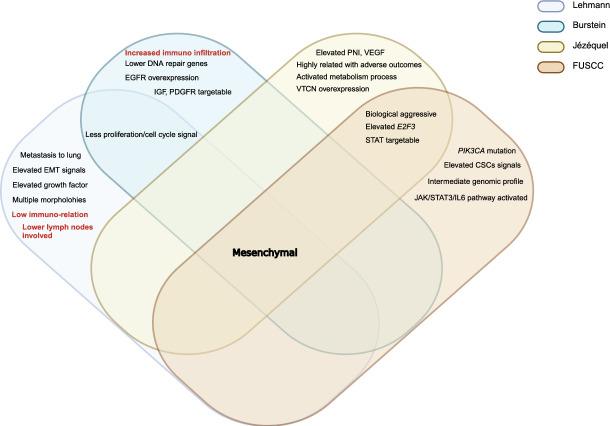
Consistency and differences in MES characteristics. Venn diagram shows the consistency and differences of four TNBC classification systems regarding Mesenchymal subtype characteristics. Controversial points for the typologies are highlighted in bold brown

We identified four main TNBC categories: the LAR subtype, which had the highest consensus, the immune subtypes including immune-active subtype and immune-suppressive subtype, as well as the mesenchymal-related subtype. These subtypes were classified based on molecular features, AR positivity status, and luminal cytologic morphology. Notably, ERBB2 gene expression was observed in Jézéquel and FUSCC classifications, even in cases that were IHC-negativity. The proliferative capacity of LAR-type cells was debated, showing reduced cell-cycle signals in the Burstein classification while exhibiting more active signals in the FUSCC classification. Generally, patients with the LAR subtype were significantly older and most of them had PIK3CA mutations (Fig. 3). The immune subtypes were classified based on immune characteristics, with most immune-active cells being BL. The Lehmann classification uniquely categorized them into BL1 and BL2 (TNBCtype-4), rather than active and suppressive types. All active subtypes demonstrated elevated immune checkpoints (PD-1, PD-L1, CTLA-4), a high TILs score, and enhanced T/B cell signals, correlating with better prognosis and outcomes. Conversely, the immune-suppressive subtype showed a significant immune enrichment score but had reduced T/B/NK cell signaling and antigen-presenting capacity, along with higher tumor grade, increased metabolic and proliferative signaling, a relatively unstable genome, and a worse prognosis. Jézéquel was the first to propose the M2/M1 ratio criterion and recommended anti-neurogenesis therapies. The FUSCC classification highlighted the presence of liver and brain metastases (Figs. 4 and 5). Mesenchymal typing was complex, existing across multiple subtypes, characterized by diverse morphologies, EMT-CSC transformation, reduced cell cycle and proliferation, significantly elevated single checkpoints (EGFR, VEGF, IGF, DOGFR), and increased aggressiveness, positioning it between BL and Luminal-like subtypes (Fig. 6).

However, these classifications did not effectively predict tolerance, sensitivity to second-line drugs, or side effects that may arise from modulating aberrant pathway models in patients of each subtype. Consequently, data collection and analysis on these factors need to be further accumulated to enhance the understanding of the constraints of the subtyping approach. To address these limitations and improve patient outcomes, future research should aim at identifying more effective biomarkers or enriching biomarker profiling, and simplifying the experimental methods for TNBC subtyping. The development of classification towards meeting individualized treatment and improve patient outcomes still requires a larger TNBC patient database. Therefore, clinical trial designs should consider evolving subtyping information, side effects, and adverse effects to better elucidate the heterogeneity of TNBC.

## Single biomarkers for TNBC profiling and therapeutics

PD-1 and PD-L1 are well established biomarkers in tumor therapies, primarily utilized to predict prognosis and clinical outcomes. Incorporating PD-L1 testing into current diagnostic workflows allows for the classification of patients’ subgroups that may benefit from anti-PD-1/PD-L1 treatment. Atezolizumab (an anti-PD-L1 antibody) and pembrolizumab (an anti-PD-1 antibody) are two FDA-approved drugs for the treatment of TNBC. In patients with early-stage TNBC, neoadjuvant treatment with atezolizumab in combination with nab-paclitaxel and anthracycline-based chemotherapy has been shown to significantly enhance pCR in those with PD-L1-positive tumors, based on the promising results from IMpassion130 clinical trial. PD-L1-positivity is defined as PD-L1-expressing tumor-infiltrating immune cells covering at least 1% of the tumor area [56], and thus neoadjuvant atezolizumab combined with chemotherapy is recommended for these patients [57, 58]. For pembrolizumab, the combined positive score (CPS), which quantifies the number of PD-L1-staining cells, is used to assess PD⁠-⁠L1 expression in TNBC patients. In phase III KEYNOTE-355 trial, patients with advance TNBC whose tumors expressed PD-L1 with a CPS ≥ 10 experienced significantly longer OS when treated with pembrolizumab plus compared to chemotherapy alone. Consequently, a CPS of ≥ 10 has been established as an appropriate criterion for recommending pembrolizumab in combination with chemotherapy for patients with advanced TNBC [59]. On the other hand, the FDA has approved pembrolizumab in combination with chemotherapy for high-risk early-stage TNBC patients, irrespective of tumor PD-L1 expression, based on the promising results from the KEYNOTE-522 trial [60].

The discovery of *BRCA* genes marks a significant advancement in the field of cancer genetics. The *BRCA* gene family consists of *BRCA1* and *BRCA2*, both of which serve as tumor suppressor genes involves in DNA repair [61]. Mutations in these genes lead to HRD, which has important implications for the treatment of TNBC [62]. Approximately 10% to 20% of TNBC cases exhibit *BRCA* mutations, with *BRCA1* mutations being particularly associated with a high lifetime risk of developing breast cancer [63]. Almost all patients with BL TNBC have been found to carry *BRCA1* mutation [64]. Therefore, considering *BRCA* status when classifying TNBC is essential for developing effective treatment strategies. The U.S. FDA has approved two PARP inhibitors, Olaparib [65] and Talazoparib [66], for use in *BRCA*-mutated TNBC. Both drugs have demonstrated prolonged OS, PFS in patients with HER-2 negative, *gBRCA* mutated tumors, highlighting their potential effectiveness in treating TNBC. The efficacy of Olaparib and Talazoparib in TNBC is still being studied. In PETREMAC trial, the respond rate of Olaparib in *BRCA* mutation carriers was 88.9%, compared to 28.6% without mutations, with no significant chemotherapy toxicities reported [67]. Additionally, early-stage patients with g*BRCA1* or g*BRCA2* mutations who underwent neoadjuvant chemotherapy experienced an average tumor volume reduction of 78% (range: 30–98%) [68].

AR is a nuclear receptor that primarily functions as a DNA-binding transcription factor to regulate gene expression [69]. AR expression is observed in approximately 20%–30% of TNBC patients. There has been a viewpoint suggesting that TNBC can be further subclassified, with the AR considered as the fourth receptor in this classification, known as quadruple negative breast cancer (QNBC) [44]. Comprehensive gene expression profiling has identified a distinct molecular subtype of TNBC characterized by AR expression. AR-positive TNBC predominantly exhibits a reduced rate of proliferation, improved disease-free survival, lower nodal metastatic rate, and older age at diagnosis, which are similar to LAR subtype [62]. It also shows increased chemoresistance and overactivation of the PI3K pathway [70]. AR positivity is tested evaluated in some patients by IHC measurement. However, there is currently no standard scoring method for AR, with different recommend cut-off value varied from 1 to 10% [44]. In a phase II clinical trial patients received enzalutamide (ENZA), an AR inhibitor, had a higher CBR at 16 weeks (33% verse 25%), longer mPFS (3.3 months verse 2.9 months) and mOS (17.6 months verse 12.7 months) [71]. In adjuvant therapy, ENZA combined with a PI3K inhibitor showed a CRB of 35% and a higher mPFS with 4.6 months in AR positive mTNBC compared to 2 months in AR negative mTNBC [42]. Enobosarm (an AR-targeted drug) combined Pembrolizumab for treating mTNBC demonstrated an OS with 25.5 months [41]. In neoadjuvant chemotherapy, the Arness trial initially highlights the effectiveness of ENZA plus paclitaxel against TNBC [72].

Trophoblast cell-surface antigen-2 (TROP-2) is a cell-surface glycoprotein that plays multiple roles in cellular functions, including the regulation of cytoplasmic Ca^2+^ levels. Due to its high expression in multilayered epithelial tissues and trophoblast cells, TROP-2 overexpression has been consistently linked to various type of tumors, making it a significant biomarker associated with tumor aggressiveness and poor prognosis [73]. In TNBC, TROP-2 is associated with tumor progression, and its overexpression is linked to increased malignancy and a higher likelihood of metastasis [74]. Approximately 86% of TNBC patients display TROP-2 positivity [75]. A notable example of anti-TROP2 therapy is Sacituzumab govitecan (SG), a Trop-2-directed antibody and topoisomerase inhibitor conjugate, which was approved in 2021 for the treatment of mTNBC [76–78]. In Phase I and II clinical trials, 108 heavily pre-treated mTNBC patients treated with SG reported an ORR of 33%, a CBR of 45%, a PFS of 5.5 months, and a median OS of 13.0 months, indicating its outstanding performance [79]. In the Phase III ASCENT study, SG demonstrated comparable efficacy, with 468 pre-treated patients showing a median PFS of 5.6 months compared to 1.7 months using the treatment of physician’s choice (TPC). Additionally, the median OS was 12.1 months versus 6.7 months, along with a manageable safety profile. Notably, this trial also included patients who did not initially have a diagnosis of TNBC [79, 80]. As of December 2022, there have been 19 ongoing or completed clinical trials involving SG [81], highlighting the unparalleled future applications and prospects of Trop-2 inhibitor in the treatment of TNBC.

Single biomarkers for TNBC profiling and therapeutics often provide limited insights into disease progression and treatment outcomes compared to systematic classifications and therapies. This highlights the necessity of pursuing straightforward methodologies that preserve accuracy to facilitate the identification of reliable biomarkers.

## Conclusion

Systematic classification reveals important molecular characteristics across various domains, including genes, proteins, biological pathways, RNA transcripts, metabolites, and immune responses. This comprehensive approach facilitates the subclassification of TNBC by identifying mutations, dysregulated pathways, and potential therapeutic targets. As a result, it fosters more personalized treatment options for TNBC patients and improves the effectiveness of therapies for specific subtypes. Despite these advantages, the practical application of systematic classification in clinical settings is still limited, with many approaches remaining theoretical, and standard chemotherapy continues to be the primary treatment. Future efforts should focus on advanced methodologies for biomarker identification and quantification, as well as developing novel targeted therapies for TNBC, to address the current ongoing challenges in TNBC management.

## Data Availability

Not applicable.

## Abbreviations

AC: Doxorubicin and cyclophosphamide
AR: Androgen receptor
BL: Basal-like
BL1: Basal like 1
BL2: Basal like 2
BLIA: Basal-like immune-activated
BLIS: Basal-like immunosuppressed
C1: Cluster 1
C2: Cluster 2
C3: Cluster 3
CBR: Clinical benefit rate
CPS: Combined positive score
CSCO: Chinese Society of Clinical Oncology
CSCs: Cancer stem cells
EFS: Event-free survival
EMT: Epithelial–mesenchymal transition
ENZA: Enzalutamide
ER: Estrogen receptor
ESMO: European Society for Medical Oncology
FUSCC: The Fudan University Shanghai Cancer Center
GO: Gene ontology
*gBRCA*: Germline *BRC*A
HRD: Homologous recombination deficiency
IGF-1: Insulin-like growth factor 1
IHC: Immunohistochemistry
IM: Immunoregulation
LAR: Luminal androgen receptor
M (Lehmann): Mesenchymal
MES (Burstein): Mesenchymal
MRI: Magnetic resonance imaging
MSL: Mesenchymal stem-like
mTNBC: Metastatic triple negative breast cancer
mTOR: Mammalian target of rapamycin
NAAG: *N*-Acetyl-aspartyl-glutamate
NCCN: National Comprehensive Cancer Network
NK: Natural killer
OS: Overall survival
pCR: Pathological complete response
PI3K: Phosphoinositide 3-kinases
QNBC: Quadruple negative breast cancer
RFS: Relapse-free survival
S1P: Sphingosine-1-phosphate
SG: Sacituzumab govitecan
SOX: SRY-box
TILs: Tumor infiltrating lymphocyte
TNBC: Triple negative breast cancer
TNF: Tumor necrosis factor
TROP-2: Trophoblast cell-surface antigen-2
WGS: Whole-genome sequencing
WHO: World Health Organization
